# Downbeat nystagmus: a practical approach based on a clinical case

**DOI:** 10.1055/s-0046-1827165

**Published:** 2026-08-13

**Authors:** Rebecca Ranzani Martins, Cristiana Borges Pereira

**Affiliations:** 1 Universidade de São Paulo, Faculdade de Medicina, Hospital das Clínicas, Departamento de Neurologia, Grupo de Distúrbios Vestibulares e do Equilíbrio, São Paulo SP, Brazil.

**Keywords:** Otoneurology, Downbeat Nystagmus, Central Vertigo

## Abstract

Spontaneous downbeat nystagmus (DBN) is the most common form of acquired nystagmus, characterized by a slow upward ocular drift and a corrective downward quick phase. The pathophysiology involves a final common pathway leading to decreased inhibition of the superior vestibular nucleus, often secondary to lesions in the flocculonodular lobes (flocculus/paraflocculus, FL/PFL), the paramedian tract (PMT), or the dorsal vermis. The clinical presentation generally includes chronic dizziness, gait unsteadiness, and oscillopsia. The causes are diverse, encompassing Chiari malformation, vascular and demyelinating lesions, hereditary and acquired cerebellar ataxias, and toxic-metabolic causes. Approximately 40% of DBN cases remain idiopathic. The treatment involves disease-specific management, prism glasses, and vestibular rehabilitation. Pharmacotherapy, such as clonazepam, gabapentin, or baclofen (with 4-aminopyridine being the most beneficial but often unavailable), is considered a second-line intervention.

## TEACHING POINTS

1. Downbeat nystagmus (DBN) is a sign of central vertigo, associated with lesions in the cerebellar vermis, midline brainstem, or the floccular/parafloccular (FL/PFL) cerebellar lobes.
2. Downbeat nystagmus is caused by cervicomedullary junction lesions, cerebellar pathologies (vascular, infectious, autoimmune/inflammatory, degenerative, genetic), nutritional deficiencies, and toxic exposures.
3. The treatment options for DBN may include optic prisms and vestibular rehabilitation, besides the cause-specific treatment.

## CLINICAL VIGNETTE

We herein present the case of a 51-year-old man with an insidious onset and progressive evolution of dizziness for 10 years, described as a constant rotatory sensation, worsening in orthostasis or while walking. At the same time, he described progressive weakness in the left side, vertical binocular diplopia, dysphonia, and dysphagia.

A neurological examination showed left hemiparesis with diminished reflexes in the upper limbs and increased reflexes in the lower limbs; superficial hypoesthesia in the thorax, neck, and upper limbs; appendicular and axial ataxias; central-pattern loss of sensation in the left face; asymmetric palatal elevation; and left shoulder drop. Eye-movement assessment revealed microsaccadic pursuit, skew deviation, and spontaneous DBN. A video of the eye-movement assessment is provided in the Supplementary Material (available at https://www.arquivosdeneuropsiquiatria.org/wp-content/uploads/2026/05/ANP-2026.0115-Video.mp4 ).

**Figure 1:**
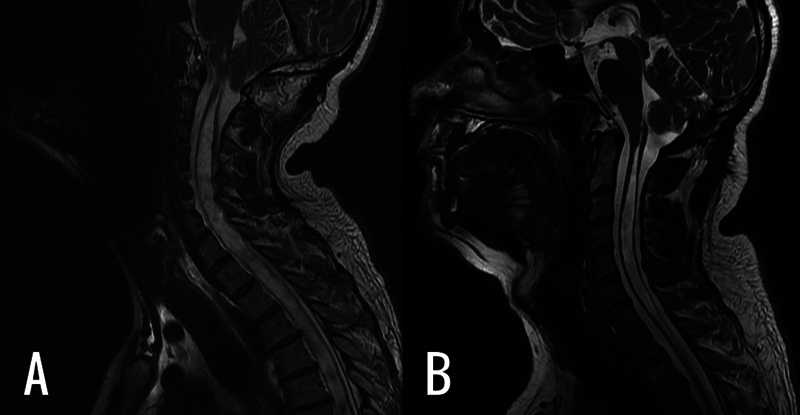
Brain and cervical magnetic resonance imaging (MRI) scan showing herniation of the cerebellar tonsils through the foramen magnum and dilation of the central canal of the spine associated with syringomyelia, both findings of Chiari type I malformation, which is classically related to spontaneous downbeat nystagmus (DBN). ( **A** ) Preoperative care. ( **B** ) After posterior fossa decompression surgery.

These findings pointed to a lesion in the brainstem and cervical spine transition. Therefore, a magnetic resonance imaging (MRI) scan showed cerebellar tonsilla herniation and cervical syringomyelia, compatible with a diagnosis of Chiari type I malformation ( Figure 1 ). The patient underwent posterior-fossa craniectomy with improvement of motor and sensory symptoms. The chronic dizziness and the DBN remained after surgery; however, these symptoms are controlled with physical therapy.

## FROM PRESENTATION TO RESOLUTION: LESSONS LEARNED

### What is the clinical presentation of downbeat nystagmus?

Spontaneous DBN is the most common form of acquired nystagmus, and it is characterized by a slow upward phase with a corrective downward phase. 1 It is observed in primary eye position and typically worsens when looking down and improves when looking up (Alexander's law). 1 In eccentric gaze, nystagmus can have a horizontal component in the same direction of the gaze; a mixed downbeat and gaze-evoked nystagmus occurs when a cerebellar lesion is involved (side-pocket phenomenon). 2

Since DBN may occur due to lesions to the cerebellar or cerebellar pathways, the ocular-motility examination can show other cerebellar signs, such as saccadic pursuit, dysmetric or slowed saccades. For the same reason, ataxic gait, limbic ataxia, and dysarthria can also be found. 3 When DBN has a structural brainstem etiology, skew deviation and/or internuclear ophthalmoplegia can be noted. 4

Patients with DBN present with blurred vision, oscillopsia (illusion of environmental motion), gait imbalance, and increased risk of falls. These symptoms are usually constant, rather than episodic. 4

### What is the pathophysiology of downbeat nystagmus?

**Figure 2:**
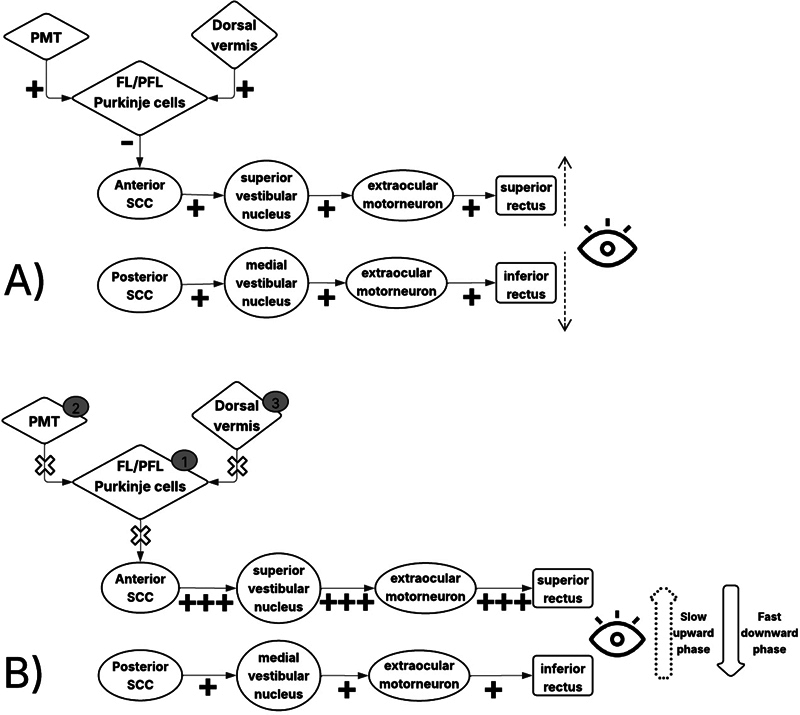
Abbreviations: PMT: paramedian tract; FL/PFL: floccular/parafloccular; SCC: superior semicircular channel. ( **A** ) Under physiological conditions, preponderance of upward eye movement, mediated by the anterior semicircular channel (SCC) and the superior vestibular nucleus, is counterbalanced by central inhibitory input from floccular/parafloccular (FL/PFL) Purkinje cells. 4 ( **B** ) Increased activity of the superior vestibular nucleus (SVN) creates a slow upward movement of the eyes, counterbalanced by a fast downward movement that constitutes DBN. The SVN is inhibited by Purkinje cells of the FL/PFL cerebellar lobes. Thus, lesions directly affecting the FL/PFL can cause DBN ( **2B.1** ). Neurons of the paramedian tract (PMT) stimulate the Purkinje cells of the FL/PFL. Therefore, midline pons-medulla lesions can damage the PMT and, in turn, decrease the function of the FL/PFL, creating a DBN ( **2B.2** ). A third possible location for SPN is the dorsal vermis ( **2B.3** ). Lesions to the dorsal vermis can impair the smooth pursuit pathway and, in turn, cause hypofunction of the FL/PFL, which is associated with DBN. Another possible explanation is that lesions of the dorsal vermis, due to direct damage to its Purkinje cells, can lead to DBN without involvement of the FL/PFL.

It has been proposed that DBN has a common final mechanism that reduces inhibition of the superior vestibular nucleus (SVN). 5 6 This increases the activity of the superior rectus muscle, creating an upward movement that is the slow phase of DBN ( Figure 2 ). Decreased inhibition of the SVN is achieved by lesions to the FL/PFL cerebellar lobes ( Figure 2B.1 ), to the paramedian tract in the midline pons and medulla ( Figure 2B.2 ), and to the dorsal vermis ( Figure 2B.3 ).

### What causes downbeat nystagmus?

It should be noted that a substantial proportion of DBN cases remains idiopathic despite investigation (up to 28.5% in combined data 1 ). Idiopathic DBN can be further divided into ‘pure’ DBN, ‘cerebellar’ DBN (associated with limb ataxia, dysarthria, and smooth pursuit impairment), and ‘syndromic’ DBN (associated with at least two of the following: bilateral vestibulopathy, peripheral neuropathy, and cerebellar signs). 1 The association of DBN with vestibulopathy and peripheral neuropathy suggests a common pathogenesis. 1 4

The causes of DBN can be divided into structurally-evident and non-structurally-evident. 1 After history taking and the neurological examination, a brain and cervical MRI scan is typically the first step in the investigation.

Downbeat nystagmus is strongly related to pathologies of the cervicomedullary junction. An important cause is Chiari malformation, which is characterized by herniation of the cerebellar tonsils below the foramen magnum. 1 7 8 Chiari type I malformation is the most commonly associated with DBN, and it can be concomitant with syringomyelia. The patients are usually young adults and present with vertigo, dizziness, and/or imbalance worsening when standing or walking, occipital headaches that also worsen in the upright position, cerebellar signs, cranial neuropathies, and sensory and motor disturbances due to syringomyelia.

Vascular 3 and demyelinating 9 lesions to the aforementioned locations are also causes of DBN. Among cerebellar pathologies, multiple-system atrophy (MSA), spinocerebellar ataxia (SCA, particularly type 6), and idiopathic cerebellar degeneration (including sporadic adult-onset ataxia) are associated with DBN. 1 3

With a normal MRI scan, subsequent diagnostic testing may be prioritized according to symptom duration and the probable underlying (such as infectious, inflammatory, genetic etc) mechanism. A list of etiologies is organized in Table 1 **,** and a diagnostic algorithm is proposed in Figure 3 .

**Abbreviations:Figure 3:**
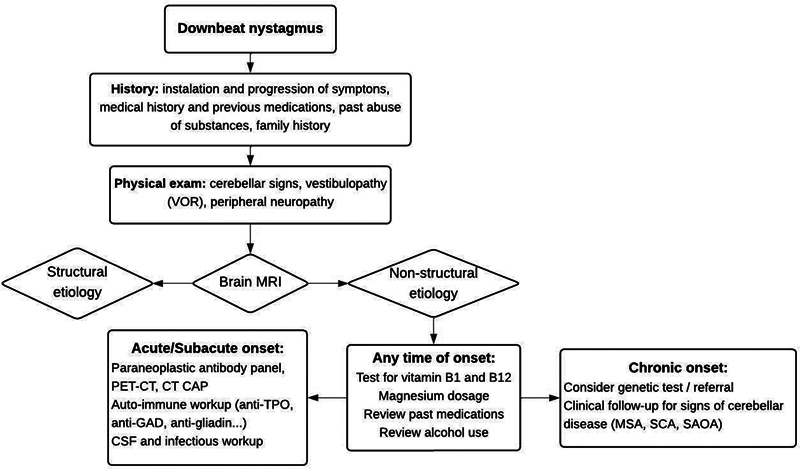
anti-GAD, glutamic acid decarboxylase antibody; anti-TPO, thyroperoxidase antibody; CAP CT: chest-abdominal-pelvis computed tomography scan; CSF, cerebrospinal fluid; MSA, multiple-system atrophy; PET-CT, positron-emission tomography-computed tomography; SCA, spinocerebellar ataxia; SAOA, sporadic adult-onset ataxia; VOR, vestibulo-ocular reflex. Diagnostic workup for downbeat nystagmus.

**Table 1:** Causes of downbeat nystagmus classified by the presence of neuroimaging findings and chronicity

Specific neuroimaging findings	Chiari malformation	
	Cervicomedullary junction lesions		
	Demyelinating lesions		
	Ischemic and hemorrhagic stroke		
	Dolichoectasia of the vertebrobasilar circulation		
Non-specific neuroimaging findings	Acute/Subacute	Paraneoplastic syndrome
		Glutamic acid decarboxylase antibody
		Celiac disease
		Hashimoto's disease
		Infectious diseases (herpes, Epstein-Barr virus, varicella, West-Nile virus, tetanus)
	Chronic	Idiopathic cerebellar degeneration
		Multiple-system atrophy
		Spinocerebellar ataxias
		Human T-lymphotropic virus infection
		Alcohol use
	Any chronicity	Drugs (antiepileptic drugs, lithium, amiodarone, intravenous opioids)
		Vitamin B1 deficiency, Vitamin B12 deficiency

Among the inflammatory and autoimmune causes are gluten-related ataxia, Hashimoto's disease (thyroglobulin and thyroperoxidase antibodies), glutamic acid decarboxylase antibody (anti-GAD)-related ataxia, paraneoplastic syndromes with ataxia (such as anti-Yo-related syndrome associated with ovarian and breast cancers). 1 4 Herpes, varicella, the West-Nile virus, the Epstein-Barr virus (EBV), tetanus, and the Human T-lymphotropic virus (HTLV) have been described as infectious causes of DBN.

Vitamin B12 deficiency should be screened in patients on a vegan diet, with a history of gastrointestinal surgery, or a history of gastrointestinal malabsorption. Vitamin B1 (thiamine) deficiency should be suspected in patients with a history of malnutrition, etilism, or pregnancy. 1 4 Interestingly, in Wernicke disease (vitamin B1 deficiency), the patients may present upbeat nystagmus in the acute phase that later converts to DBN. 10 Hypomagnesemia can occur in conjunction with vitamin B1 deficiency or isolated. 1 4

Cerebellar ataxia, neuropathy, and vestibular arreflexia syndrome (CANVAS) is a recently-described entity associated with DBN, cerebellar ataxia, peripheral neuropathy, bilateral vestibulopathy, and chronic cough. 11 It is caused by a biallelic repeat expansion in an intronic region of the *RFC1* gene.

The workup should also include a history of prior medications and substance use. Lithium is a cause of DBN, and nystagmus may persist after the cessation of the therapy. Downbeat nystagmus has also been reported with antiepileptic drugs (AEDs), such as phenithoin and carbamazepine, intravenous opioids (morphine, phentanyl), amiodarone, and metronidazole. 1 3 4 Chronic alcohol consumption, with or without thiamine deficiency, is another possible cause of DBN. Neuroimaging may present cerebellar atrophy, but it is not mandatory. 1 3 4

### How to treat downbeat nystagmus

The treatment of DBN includes disease-specific management, such as thiamine replacement for Wernicke encephalopathy or surgical management for Chiari malformation.

Regardless of the cause, DBN symptoms can be improved with prism glasses and physical therapy (focused on vestibular rehabilitation). Many DBN patients adopt a compensatory head posture to place their eyes in a position that minimizes nystagmus, called the *null point* . Prism glasses can redirect the visual field towards the null point, so the patient can adopt a more comfortable head position. By operating in a gaze which reduces nystagmus, patients can also report milder oscilopsia. 1 12 Another interesting observation that can be used to guide patient counseling is that DBN is usually worse in the morning and improves during the day, in the dark, and while resting in an upright position. 1 4 These observations can guide daily-life recommendations for patients.

Pharmacotherapy is a second-line intervention and is more beneficial when used concomitantly with physical therapy. 4-aminopyridine (4-AP) and 3,4-diaminopyridine (3,4-AP) show the most benefit in DBN, but they are unavailable in Brazil. Clonazepam, gabapentin, and baclofen are alternatives. 1 12

Ultimately, DBN is a neuro-ophthalmologic sign with a wide and heterogeneous differential diagnosis, from structural cerebellar or cervicomedullary lesions to metabolic, immune-mediated, toxic, and degenerative disorders. A careful history taking, physical examination, and targeted investigation are essential to identify treatable causes and guide the appropriate management. Although many cases remain idiopathic, understanding the disease and its associated conditions enables neurologists to optimize disease-specific therapy and symptomatic treatment, improving the functional outcomes of the patients.
